# Bioactive Polyketides from *Amphidinium* spp.: An In-Depth Review of Biosynthesis, Applications, and Current Research Trends

**DOI:** 10.3390/md23060255

**Published:** 2025-06-16

**Authors:** Noemi Russo, Giulia Quaini, Marcello Ziaco, Daniela Castiglia, Alessandra Ruggiero, Vincenzo D’Amelia, Concetta Di Napoli, Sergio Esposito, Angelo Fontana, Genoveffa Nuzzo, Simone Landi

**Affiliations:** 1 Department of Biology, University of Naples Federico II, Via Cintia 21, 80126 Naples, Italy; noemi.russo@unina.it (N.R.); giulia.quaini@unina.it (G.Q.); concetta.dinapoli2@unina.it (C.D.N.); esposito@unina.it (S.E.); angelo.fontana@cnr.it (A.F.); 2 Bio-Organic Chemistry Unit, Institute of Biomolecular Chemistry, National Research Council, Via Campi Flegrei 34, 80078 Pozzuoli, Italy; m.ziaco@icb.cnr.it (M.Z.); daniela.castiglia@cnr.it (D.C.); 3 Institute of Bioscience and Bioresources, National Research Council, Via Università 133, 80055 Portici, Italy; alessandra.ruggiero@cnr.it; 4 Department of Agronomy, University of Naples Federico II, Via Università 100, 80055 Portici, Italy; vincenzo.damelia2@unina.it

**Keywords:** marine natural products, polyketides, polyketide synthases, dinoflagellates, *Amphidinium* spp.

## Abstract

Polyketides (PKs) are a widespread class of secondary metabolites with recognised pharmacological properties. These molecules are abundantly produced in the marine environment, especially by dinoflagellate-photosynthetic organisms able to produce several PKs, including neurotoxins, cytotoxins, and immunomodulating agents. The biosynthesis of these compounds is driven by a conserved enzymatic process involving polyketide synthase complexes. Different genera of dinoflagellates produce PKs. Among them, dinoflagellates of the genus *Amphidinium* are of particular interest due to its ability to produce the following two major families of PKs: amphidinolides and amphidinols. These compounds display remarkable biological activities, including anticancer, antimicrobial, and antifungal effects, making them attractive targets for pharmaceutical research and development. However, the natural yield of *Amphidinium*-derived polyketides (APKs) is generally low, limiting their potential for sustainable molecular farming. This challenge has prompted interest in developing biotechnological strategies to enhance their production. This review aims to define the current state of studies about APKs, starting from their initial discoveries to the recent understanding of their biosynthetic pathways. Additionally, it summarizes the structures of compounds discovered, highlights their biotechnological potential, and discusses novel trends in their production.

## 1. Introduction

The increased demand for novel compounds for biotechnological applications has significantly intensified the research of new natural compounds in the marine environment [1,2]. Through a combination of bioassay-guided isolations and novel omics approaches, numerous Marine Natural Products (MNPs) have been identified [3]. According to the MarinLit database (January 2025), a total of 42.652 MNPs have been isolated from sponges, algae, microbes, corals, and sea cucumbers [2,4,5]. The global market value for these products is predicted to reach USD 9,275.9 million by 2034 [6]. These compounds showed remarkable biological properties, such as antibacterial, antifungal, immunomodulant, antiviral, and anticancer activities, and some of them have inspired the development of synthetic analogues [7,8,9,10,11]**.** Currently, fifteen marine-derived drugs are available in the pharmaceutical market (e.g., Epanova^®^, Yondelis^®^), ten of which are used for cancer treatment, while others are actually involved in clinical trials [12].

Among the marine-derived products, polyketides (PKs) showed a wide range of biological activities, emphasising their importance in drug discovery and development. This is further evidenced by the growing interest the scientific community has for these molecules. PKs are a rich class of secondary metabolites, both in terms of structural diversity and biological activity. They are usually characterised by highly oxygenated skeletons, occasionally organised in macrocyclic lactones, with an elevated number of stereocenters. Furthermore, a wide range of molecule decorations (e.g., glycosylations, epoxidations, and other modifications, such as halogenation) can highly diversify the structure of PKs, generating compounds with different molecular weights, ranging from 0.3 to 2.6 kDa, and even larger in some exceptional cases [13,14,15].

PKs are produced by different marine organisms. Among these, dinoflagellates represent a peculiar and widespread class of planktonic unicellular protists belonging to the Alveolate group [16,17]. The number of species comprised in these taxa was recently estimated at around 6000 species [18,19]. Dinoflagellates are important primary producers in microplankton communities; indeed, 51% of these species exhibit photosynthetic capabilities. Their extensive genetic and biochemical diversity enables the production of a wide range of PKs. Among dinoflagellates, the genus *Amphidinium* is particularly noteworthy in producing bioactive compounds. Nowadays, over a hundred structurally unique molecules have been identified from *Amphidinium* species [20,21]. Many of these are PK-derived molecules, including both linear and macrocyclic structures, such as amphidinins, amphirionins, amphidinols, amphidinolides, iriomoteolides, and other related compounds. Despite the biological potential of these molecules, their pharmaceutical exploitation remains limited due to the low productivity of dinoflagellates and difficulties related to their culturing on laboratory, pilot, and industrial scales [1]. Other attempts involve biological chemistry strategies to overcome the supply limitation of natural products for clinical development. However, this can be highly challenging due to the structural complexity and stereochemistry of molecules. A straightforward approach involved the use of synthetic biology to boost their production. A crucial step in this process is the identification of biosynthetic gene clusters encoding for enzymatic complexes present in the genomes of these microorganisms.

This review aims to provide a comprehensive evaluation of the current state of *Amphidinium* polyketides (APKs) research, from their discovery to their structural diversity and potential applications. Specific emphasis will be placed on their biosynthetic pathways, biotechnological potential, and emerging trends in their production, with the scope of assisting synthetic biologists and researchers in developing effective biotechnological strategies. Considering their significance, APKs’ molecular diversity and their systematic classification have been extensively reviewed elsewhere [17,18,19,20,21,22,23,24], and will therefore not be discussed in detail in this review.

## 2. An Overview of Dinoflagellate Polyketides

Dinoflagellates possess some of the largest known genomes among eukaryotes, with sizes up to 80 times that of the human haploid genome [22,23]. Throughout evolution, these genomes have assimilated genetic material from different sources, including the peridinin plastid, tertiary replacement plastid, cyanobacteria, red algae, green algae, haptophytes, and even bacteria [22]. This extensive genetic integration has resulted in highly chimeric nuclear genomes, enabling dinoflagellates to synthesise a vast array of bioactive compounds [24,25]. These compounds often exhibit slight variations in their functional groups, presenting significant opportunities for drug discovery and biotechnological innovation [24].

Dinoflagellate PKs include a huge and diverse set of molecules, both as chemical structures and biological activities. These products are often highly oxygenated and comprise macrolides, polyethers, polyols, and aromatics [14]. Some dinoflagellate PKs are considered neurotoxic. In particular, they are classified into the following five major categories: paralytic shellfish poisoning (PSP), neurotoxic shellfish poisoning (NSP), amnesic shellfish poisoning (ASP), ciguatera fish poisoning (CFP), and diarrheic shellfish poisoning (DSP). The combination of their toxicity and high growth rate during eutrophication events can lead to harmful algal blooms that raise significant concerns for public health, environmental safety, and human activities [26].

Many dinoflagellate PKs were identified in recent decades. Hereinafter, a brief overview of some of the most significant molecules produced by dinoflagellates of the *Amphidinium* genus is provided.

## 3. *Amphidinium* Polyketides: Chemical Structures and Biological Activities

**Figure 1:**
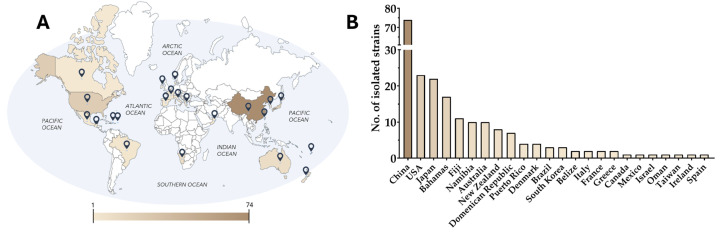
Map of countries (**A**) and number of isolated Amphidinium species (**B**) (*Amphidinium carterae*, *Amphidinium cupulatisquana*, *Amphidinium fijiensis*, *Amphidinium gibbosum*, *Amphidinium herdmanii*, *Amphidinium incoloratum*, *Amphidinium magnum*, *Amphidinium massartii*, *Amphidinium cf. massartii*, *Amphidinium mootonorum*, *Amphidinium operculatum*, *Amphidinium pseudomassartii*, *Amphidinium paucianulatum*, *Amphidinium steinii*, *Amphidinium theodori*, *Amphidinium thermaeum*, *Amphidinium cf. thermeaum*, *Amphidinium trulla*, *Amphidinium tomasii*). This figure includes 211 different strains collected from all over the world.

*Amphidinium* refers to an entire genus of dinoflagellates belonging to the novel order of *Amphidiniales* [27]. This genus includes more than 100 species, from both freshwater and marine biotopes, which have been isolated from phytoplankton, sand, coral, and macroalgae, with a pronounced biodiversity in the Atlantic and Pacific oceans (Figure 1) [28,29,30]. These athecate organisms are defined as benthic, planktonic, or endosymbiotic species [28].

Originally, their classification was based on morphological features, giving rise to inaccurate and, in some cases, ambiguous attributions [31]. The employment of molecular phylogenetic methods, encompassing the sequencing of the large-subunit (LSU) region of rDNA and internal transcribed spacer regions I and II (ITS1 and ITS2), has led to the elucidation of numerous taxonomic and phylogenetic relationships [29,32]. The classification system now groups the species with minute, irregular, triangular, or crescent-shaped epicones (deflected to the left) into *Amphidinium sensu stricto* species. The remaining species are grouped into *Amphidinium sensu lato* [20,33]. Beyond their morphology or classifications, different strains belonging to this genus, like *A. carterae*, *A. massartii*, *A. fijiensis, A. gibbosum*, and *A.operculatum,* are officially classified as toxigenic or non-toxigenic based on their ability to synthesise extremely lethal compounds associated with fish kills [20,33]. The identified APK compounds are hereby classified into the following two main groups: linear polyketides and macrolides.

### 3.1. Linear Polyketides

**Figure 2:**
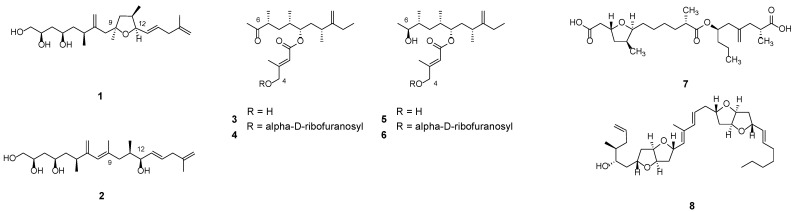
Amphidinins A (**1**), G, (**2**), C (**3**), D (**4**), E (**5**), F (**6**), B (**7**), amphirionin 2 (**8**).

Among the shorter linear compounds reported from *Amphidinium*, amphidinins (Figure 2, **1**–**6**) represent a noteworthy class of PKs. These metabolites characteristically feature a linear backbone comprising 17 carbon atoms, variously decorated with hydroxyl and methyl groups. Amphidinin A (**1**) and G (**2**) are structurally related; amphidinin G (**2**) represents a precursor of amphidinin A (**1**), as the latter is derived from the formation of the tetrahydrofuran ring through the addition of a hydroxyl group at C12 to an olefinic carbon at C9 [34]. In contrast, derivatives C–F (**3**–**6**) are structurally related to the cytotoxic 12-membered macrolide amphidinolide Q (**34**). Amphidinin derivatives have been reported to possess cytotoxic properties, as well as significant antifungal and antibacterial potential [34]. In particular, amphidinin A (**1**), C (**3**), and E (**5**) demonstrated antimicrobial activities against *Bacillus subtilis* and *Aspergillus niger*, whereas only amphidinin C and E exhibited activity against *Staphylococcus aureus* [35]. The complete set of amphidinins was found in strain 2012-7-4A, except for amphidinins A (**1**) and B (**7**), which were well-characterised in Y-5 and Y-56, respectively [35,36,37].

Recently, novel short linear PKs, amphirionins, have been identified [38,39,40,41]. Amphirionin 2 (Figure 2, **8**) is a PK consisting of a linear C30 carbon chain, and it is the first reported APK that possesses a hexa-hydro-furo [3,2-b]-furan moiety. Amphirionin 2 (**8**)’s importance is linked to its cytotoxic activities against human tumour cells, like colon and lung carcinoma [38]. In addition, amphirionin 2 was found to have *in vivo* activity against different tumours in murine models [38].

A complex and characteristic PKs family isolated from both planktonic and symbiotic Y-5 strains includes polyoxygenated long carbon chain products, such as amphidinols, luteophanols, lingshuiol, and colopsinols.

**Figure 3:**
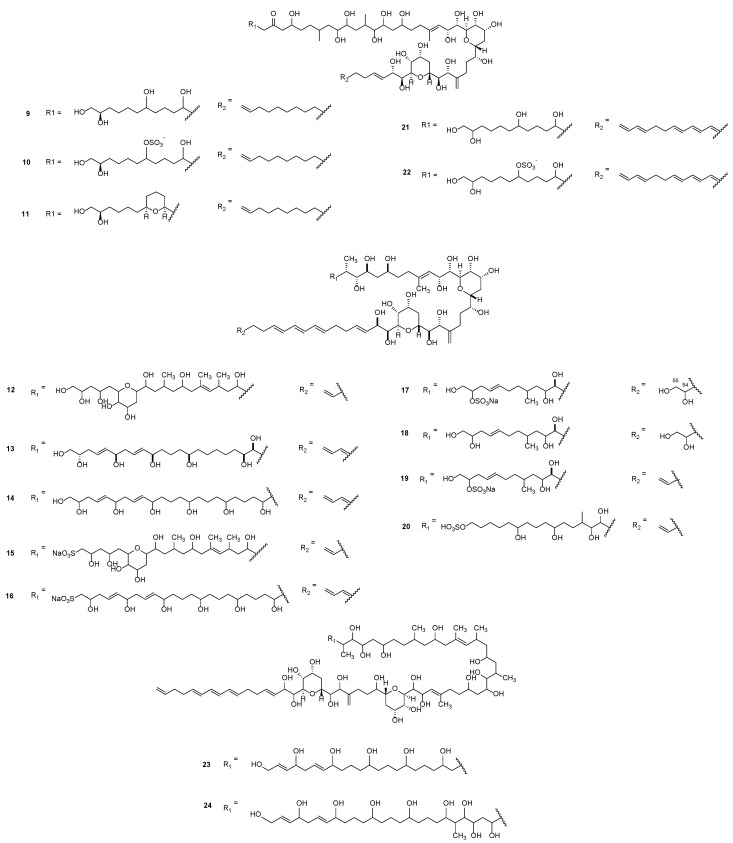
Amphidinols A (**9**), B (**10**), C (**11**), 2 (**12**), 3 (**13**), 9 (**14**), 11 (**15**), 13 (**16**), 14 (**17**), 15 (**18**), 7 (**19**), 17 (**20**), 18 (**21**), 19 (**22**), 20 (**23**), 21 (**24**).

The first identified amphidinols (AMs) were reported by *Satake* et al. [42] (Figure 3). These compounds shared common structural features characterised by a well-conserved polyunsaturated highly oxygenated linear backbone, two tetrahydropyran rings, and two distinctive side chains that differ in levels of saturation, oxidation, and substitution [43]. They can be distinguished in A to C and AM1 to 13 analogues, both of which have been shown to possess recognised potent antifungal and haemolytic activities [43,44,45,46]. AM-A (**9**) and B (**10**) were identified by a specific strain of *A. carterae,* isolated from seaweed samples collected at Lake Fusaro [45]. AM-C (**11**) was isolated from the equivalent Irish species, and its relative configuration was established based on a putative biosynthesis scheme of the new ring from a nucleophilic substitution of the sulphate that is present on the AM-B side chain [46]. A comparison of **12**–**16** suggests that sulfation at C1 resulted in a significant reduction in antifungal and haemolytic activities [43].

Two additional amphidinol homologues, AM14 (**17**) and AM15 (**18**), were isolated from cultured *Amphidinium klebsii* and structurally elucidated. These compounds are closely related to AM7 (**19**), differing only by the presence of two hydroxyl groups at positions C54 and C55 in the second lateral chain [47]. The influence of the polyhydroxyl moiety on biological activity was investigated, revealing that variations in the length of the chain had minimal impact on the pore size formed by the AM-induced membrane channels [44]. Furthermore, studies on AM17–19 (**20**–**22**) analogues indicated that sulfation negatively affects the anti-infective properties of AMs, regardless of the esterification site [48,49]. Additional novel amphidinols, featuring exceptionally long polyol chains, were characterised as AM20 (**23**) and AM21 (**24**), the latter of which was distinguished by possessing the longest linear polyol structure reported among amphidinol analogues to date [50].

**Figure 4:**
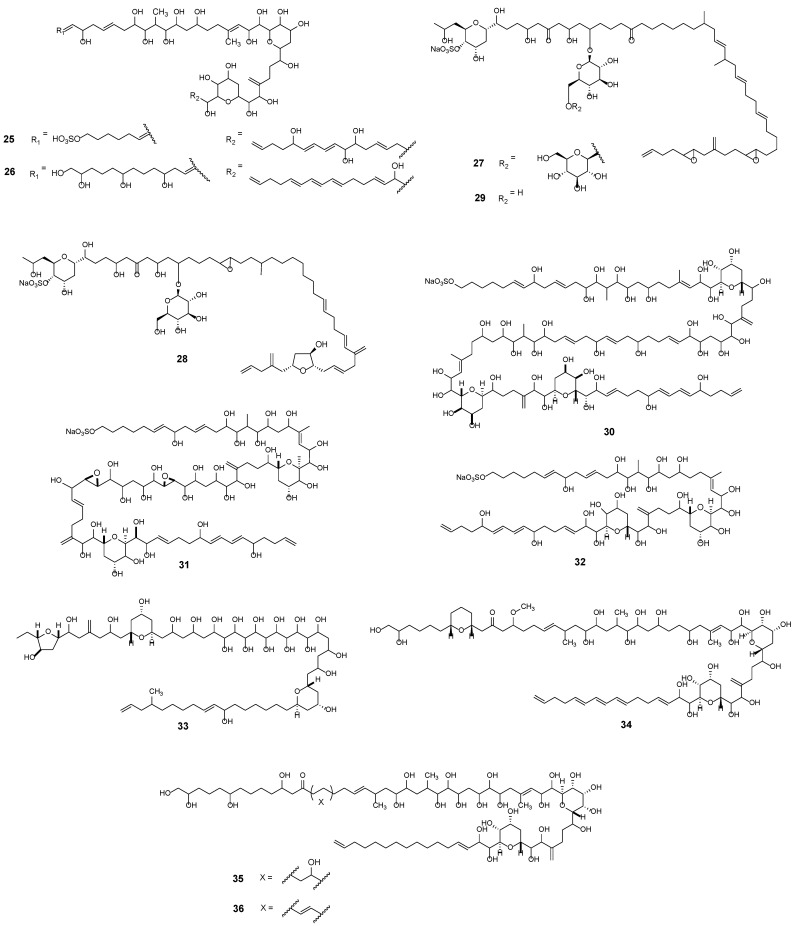
Luteophanol A (**25**), Lingshuiol A (**26**), Colopsinol A (**27**), C (**28**), E (**29**), Amdigenol A (**30**), E (**31**), G (**32**), Amphezonol A (**33**), Carteraol E (**34**), Karatungiol A (**35**), B (**36**).

Luteophanols A–D (e.g., Lut A Figure 4, **25**) from symbiotic *Amphidinium* sp. (strain Y-52) are characterised by a C57 linear aliphatic chain with one sulphate ester and two tetrahydropyran rings [51,52,53]. The configuration of pyran rings is suggested to contribute to a hairpin-like structure that enhances their membrane-disrupting properties [54]. Lingshuiols A (Figure 4, **26**) and B are polyhydroxy compounds characterised by a linear carbon chain with potent cytotoxic activity [55]. As AM homologues, they possess potential antifungal properties and permeabilising activity that is stronger than AM2 (**12**) [56].

Colopsinols A–C represent the first identified members of a novel class of polyketides (PKs) characterised by the presence of both a glucoside moiety and a sulfate ester. These compounds were isolated from strain Y-5 [57,58,59]. Within this group, structural diversity arises primarily from differences in glycosylation levels. Notably, colopsinol A (**27**) showed potent inhibitory activity against DNA polymerases α and β, whereas analogues C (**28**) and E (**29**) exhibited cytotoxicity against L1210 cells in vitro [60].

Another class of structurally analogous large polyol compounds includes amdigenols [61,62,63]. Amdigenol A (**30**) consists of two amphidinol core structures suggesting that the C98-linear carbon backbone is a dimeric form of amphidinol [61]. Amdigenols A (**30**), E (**31**), and G (**32**) have been shown to inhibit the elevation of the intracellular Ca^2+^ concentration in differentiated IMR-32 neuroblastoma cells [62]. Amphezonol A (**33**), a C60 linear polyketide, was isolated from the cultured strain Y-72, originally obtained from the acoel flatworm *Amphiscolops* sp., and displays inhibitory activity against DNA polymerase [64]. Carteraol E (**34**) (strain AC021117009) was isolated from the wash-off epiphytes of seaweed [65]. It is a C69 compound structurally related to amphezonol, differing primarily in the nature and distribution of its functional groups. It exhibits notable cytotoxicity, with an LD_50_ value of 0.28 mmol/L, along with antifungal activity against *Aspergillus niger* [65]. Lastly, karatungiols A (**35**) and B (**36**) are long chain amphidinol analogues that show significant antifungal activity against NBRC4407 *A. niger* and antiprotozoan activity against *Trichomonas foetus* [66].

### 3.2. Macrolides

**Figure 5:**
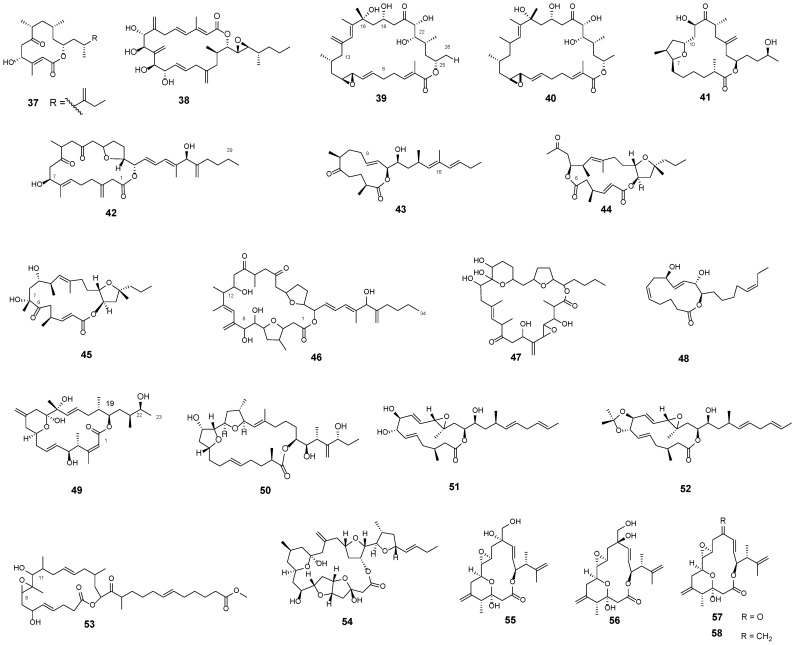
Amphidinolide Q (**37**), A (**38**), B (**39**), B1 (**40**), T2 (**41**), U (**42**), W (**43**), X (**44**), Y (**45**), C (**46**), Caribenolide I (**47**), Amphidinolactone A (**48**), Iriomoteolide 1a (**49**), 2a (**50**), 3a (**51**), 3b (**52**), 11a (**53**), 13a (**54**), 14a (**55**), 14b (**56**), Amphidinolide O (**57**), and P (**58**).

The linear PKs amphidinins are intrinsically linked to another class of metabolites synthesised by dinoflagellates, known as amphidinolides (amphidinins C–F (**3**–**6**) were 4,5-seco analogues of amphidinolides Q (**37**)). Amphidinolides (Figure 5) are classified as macrolides, a category of secondary metabolites typically characterised by a highly oxygenated polyene backbone and a macrocyclic lactone ring. Since the first identification of amphidinolide A (**38**) [67], more than 30 cytotoxic macrolides have been identified. Recent studies have expanded the amphidinolide family with the discovery of new members, including those from the B series (e.g., B, **39**; B1, **40**), T series (e.g., T2, **41**), and U–Y series (**42**–**45**), as well as the G and H series (structures not reported). Among this diverse group, amphidinolides A–S (e.g., A, **38**) [35,67,68,69,70,71,72,73,74,75,76,77,78] have demonstrated notable anticancer activity against various cancer cell lines, including human colon carcinoma, murine leukaemia L1210, and KB human epidermoid carcinoma. Notably, amphidinolides B1–B4 (e.g., B1, **40**) have demonstrated potent cytotoxicity at nanomolar concentrations against human tumour cell lines, with marked selectivity toward human colorectal carcinoma cells [79]. The amphidinolide T series, comprising compounds T2–T5 (e.g., T2, **41**) [76,80], represents a group of 19-membered macrolides characterised by the presence of a furan ring spanning carbons C7 to C10. In addition, several structurally intriguing amphidinolides, namely U (**42**), W (**43**), X (**44**), and Y (**45**), have been isolated and structurally characterised through detailed spectroscopic analyses [81,82,83,84]. These molecules share structural motifs with previously reported amphidinolides. For instance, the C7–C29 and C1–C8 segments of amphidinolide U (**42**) are structurally analogous to the C12–C34 and C1–C8 regions of amphidinolide C (**46**) and amphidinolide A (**38**), respectively, suggesting a shared biosynthetic origin. In contrast, amphidinolides X (**44**) and Y (**45**) exhibit unique structural features. Oxidative cleavage of the C6–C7 bond in amphidinolide Y using lead tetra-acetate yields amphidinolide X, supporting the hypothesis that amphidinolide Y serves as a biogenic precursor to amphidinolide X. This transformation underscores the complex and interconnected biosynthetic pathways that give rise to these structurally diverse and biologically significant macrolides.

Caribenolide I (**47**), isolated from the *Amphidinium* strain Sl-36-5, features a macrocyclic lactone incorporating an α-methylene epoxide and a pyran ring, structurally distinguishing itself from other known macrolides. This compound exhibited approximately 100-fold greater cytotoxic activity than amphidinolide B (Figure 5, **39**) [85]. Amphidinolactone A (**48**), isolated from strain Y-25, is notable as the first reported macrolide from *Amphidinium* sp. that lacks both a branched methyl group and an exo-methylene moiety. It is a thirteen-membered macrolide composed of a C20 carbon backbone, containing four disubstituted double bonds and two hydroxyl groups, and has shown cytotoxic activity against L1210 murine leukaemia cells [86].

Iriomoteolides (Figure 5) are a recently identified class of macrolides from *Amphidinium* dinoflagellates, characterised by a potent cytotoxicity and promising antitumour activity [34]. Iriomoteolide 1a (**49**) is a cytotoxic 20-membered macrolide isolated from the strain HYA024 [87]. The first 23-membered macrolide in this series, iriomoteolide 2a (**50**), shows a continuous polyketide chain and has demonstrated in vivo antitumour activity [88]. Iriomoteolide 3a (**51**) is a rare example of a 15-membered macrolide, accompanied by its 7,8-O-isopropylidene derivative, named iriomoteolide 3b (**52**) [89]. These low-molecular-weight compounds have been shown to target the actin cytoskeleton, a critical component of numerous cellular processes, and are therefore recognised as valuable molecular probes [90]. Iriomoteolide 3a (**51**) and its analogues induce pronounced morphological alterations in the actin filament network at sub-micromolar concentrations, leading to inhibition of cell migration and cytoplasmic retraction [90].

Iriomoteolide 11a (**53**) is a unique 19-membered macrolide composed of 34 carbon atoms and distinguished by the presence of a carbomethoxy terminal group [91]. In contrast, iriomoteolide 13a (**54**) features more intricate architecture, incorporating a hexahydro-furo [3,2-b]furan ring, a tetrahydropyran ring, two tetrahydrofuran rings, three one-carbon side chains, and three hydroxyl groups, including two hemiketal functionalities [92]. Most recently, iriomoteolides 14a (**55**) and its 11R-isomer 14b (**56**) have been identified as structural analogues of amphidinolides O (**57**) and P (**58**). Both 14a and 14b exhibit cytotoxic activity against human cervix adenocarcinoma HeLa cells, with a potency comparable to that of their amphidinolide counterparts [93].

## 4. *Amphidium* Polyketides Biosynthesis

APKs are synthesised by Polyketide Synthases (PKSs), proteins that organised linearly to execute a series of sequential reactions. PKSs are composed of multiple modules that individually catalyse C-C bond formation and peculiar modification of elongated intermediates. This process shows analogies with the biosynthesis of fatty acids (FA), probably due to the shared evolution of PKSs and Fatty Acid Synthases (FASs) [94,95].

**Figure 6:**
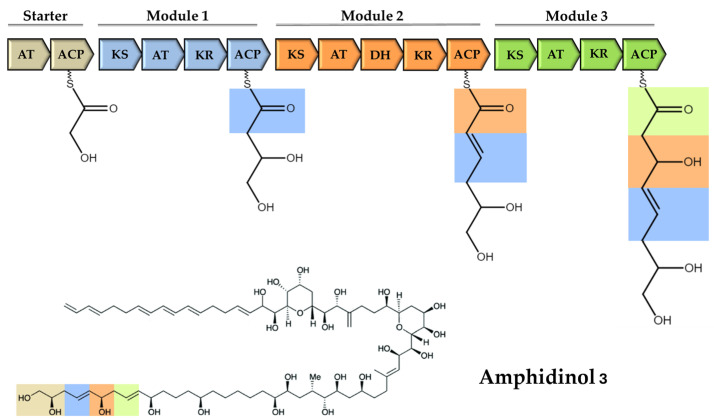
Putative biosynthesis of the initial segment of Amphidinol 3: a schematic representation of the polyhydroxy moiety elongation based on modular type I PKS. Domains are acyl transferase (AT), acyl carrier protein (ACP), ketosynthase (KS), ketoreductase (KR), and dehydratase (DH).

PKs’ biosynthesis can be divided into the following three stages: initiation, elongation/modification, and termination. This sequence involves the incorporation of carboxylic acid units onto a growing acyl chain through repeated Claisen condensations. Paradigmatically, this process begins with the activation of an acetyl-CoA starter unit by an acyl transferase domain (AT), which is then loaded onto the 4′-phosphopantetheinylated acyl carrier protein domain (ACP). The intermediate linked to ACP is then transferred to the ketosynthase domain (KS), where decarboxylative Claisen condensation occurs, linking the malonyl substrate to the growing polyketide chain [96,97]. Other domains are involved in modifying the β-keto group to a hydroxyl group (ketoreductase -KR), forming a double bond (dehydratase -DH) and completing the saturation of the acyl chain (enoylreductase -ER) [98]. At the end of the biosynthetic process, a thioesterase domain (TE) is required for the release of the chain by intramolecular cyclisation or direct hydrolysis. In particular, TE domains catalyse a transesterification step via their active-site serine, ultimately mediating the final product offloading [99]. A putative mechanism of synthesis for AM3 is represented in Figure 6.

PKSs can be classified into three main types (I, II, and III), based on their architecture. Type I are large, multifunctional enzymes composed of multiple catalytic domains, further classified into iterative and modular systems [100,101]. Iterative PKSs are single-module enzymes capable of performing repeated cycles of synthesis [102]. Modular PKSs consist of multiple modules functioning in an assembly-line manner. Each module contributes to a specific step in the biosynthetic process [103]. Furthermore, modular PKSs can be divided into cis-AT and trans-AT. Cis-AT PKSs contain all three core domains (KS, AT, and ACP) within the same module, while trans-AT PKSs lack an integrated AT domain, relying on a free-standing AT enzyme that transacylates the ACP domain [104]. Type II PKSs are made by distinct catalytic domains that iteratively operate as independent mono-domain proteins [105]. Finally, type III PKSs are simpler homodimer enzymes with a single active site operating iteratively, without the involvement of an ACP [106].

In addition to PKS, polyketide biosynthesis, particularly that containing amide bonds, is assisted by non-ribosomal peptide synthetases (NRPS). These giga-enzymes can form hybrid PKS–NRPS formations, significantly expanding the range of possible products [107]. A recent outcome in the PKS biosynthesis of marine photosynthetic organisms was the identification of giant proteins called PKZILLA-1 and -2 in *Prymnesium parvum* [108]. These are PKS of 4.7 and 3.2 megadaltons that have 140 and 99 enzyme domains involved in the biosynthesis of prymnesin, one of the largest nonpolymeric compounds in nature.

Different studies reported the presence of type I and type II PKSs in dinoflagellates, with no evidence of type III [17]. Among type I PKS, both cis- and trans-AT have been observed in dinoflagellate species. As reported by Haq et al. [109], type I PKS trans-ATs are considered the most likely candidates in the production of toxins [110]. Unlike bacterial PKs, which involve small carboxylic starter units such as propionate, butyrate, isobutyrate, valerate, and isovalerate, dinoflagellate mainly involves acetate and, in some cases, glycolate [111,112]. Moreover, dinoflagellate PKs synthesis requires additional reactions during the elongation steps or modifications post-PKS synthesis [111]. In these cases, acetate represents the primary extension unit, which can undergo cleavage processes, resulting in a carboxyl or methyl carbon unit. The biosynthesis of PKs from dinoflagellates, namely brevetoxins [113,114], okadaic acid [115], yessotoxins [116], and amphidinolides [117], involves multiple cleavage steps during their production [118,119]. For carbon deletion reactions, three hypotheses have been proposed, which are as follows: (i) carbon deletion mediated by intermediate metabolites from the tricarboxylic acid cycle; (ii) carbon deletion through Favorskii-type rearrangement; and (iii) carbon deletion via specific functional modules within PKS [120,121,122]. However, the second hypothesis is the most widely accepted [122]. Other typical dinoflagellate reactions include alkylation, such as α-alkylation, β-alkylation, and pseudo α-alkylation. A-Alkylation is catalysed by methyl transferase enzymes. β-alkylation occurs through the incorporation of pendant methyl groups derived from the methyl carbon of an acetate unit to the β-carbon. When the same methyl group is added to the α-carbon, the reaction is called pseudo α-alkylation [122,123]. Furthermore, dinoflagellate PKs may contain several ether rings (five- to nine-membered), with unique sizes and spatial configurations [111].

### 4.1. Amphidinium PKS Genes Discovery

The identification of putative PKS genes from *Amphidinium* spp. was originally provided by *Snyder* et al. [124] using PCR and RT-PCR. This investigation was based on degenerate primers designed on KS domains of bacterial type I PKSs and fungal type II PKSs, and used to screen seven dinoflagellate species, including *A. operculatum* (CCMP1342, CCMP120, CCMP121) and *A. carterae* (CCMP1314). Apart from *A. operculatum* and *A. carterae*, all of the above strains showed the presence of homologous KS domains, revealing the presence of putative PKS type I [17,124]. Similar methodologies were employed by *Kubota* et al. [125] to investigate the genomic DNA of five *Amphidinium* strains able to produce amphidinolides and eight strains unable to produce similar molecules. A total of fourteen KS domains (PKS type I) were detected in the amphidinolide-producing strains (Y-5, Y-42, Y-71, Y-72, and Y-100). Genomic DNA from *Amphidinium* sp. Y-42 was then used to construct a fosmid library. The analysis of 100,000 fosmid clones for the presence of PKS-related sequences yielded a single positive clone with an insertion fragment of 36.4 kb. The insert was entirely sequenced, revealing six ORFs homologous to KS, AT, DH, KR, ACP, and TE domains. The six ORFs, found in the middle of the sequence, accounted for only 15.5% of the total length (5625 bp). Interestingly, several frame shifts occur within and between catalytic domains, and the protein-coding region was found to be divided into two sections by a 4 kb stretch, which was, according to the authors, attributed to a putative intron sequence [17,125]. *Bachvaroff* et al. [126] proposed the classification of *A. carterae* genes into two different categories, as follows: (1) highly expressed, tandemly repeated genes with low intron density, and (2) moderately expressed, non-tandem genes with high intron density. PKS genes belong to the second category [127].

Further advancements about PKSs have been achieved through transcriptomic and biochemical analyses, which revealed the presence of non-canonical, multi-modular hybrid PKS/NRPS systems in various dinoflagellates, including *A. carterae*. This finding identified three distinct domain arrangements, two of which are similar to bacterial genes. These findings suggested a horizontal transfer between prokaryotes and dinoflagellates. One of these arrangements exhibited a strong similarity to BurA, a gene found in the *Burkholderia* species, both in sequence and domain organisation. Another arrangement showed a resemblance to ZmaK from *Bacillus cereus*. Additionally, the Triple KS transcript (known as 3KS) was identified due to the presence of three KS domains. The 3KS sequence has attracted significant attention considering its potential correlation with toxicity. However, despite various attempts to characterise it, there is no definitive evidence regarding its biosynthetic functionality [109,126,127,128,129,130,131].

### 4.2. Labelling Experiments in Amphidinium Polyketides Biosynthetic Studies

Since their isolation, the biosynthetic origin of APKs has been extensively investigated using isotope labelling experiments by NMR and MS techniques. Feeding experiments with [1-^13^C], [2-^13^C] and [1-2-^13^C]-acetate have been performed to obtain ^13^C-enriched PKs and track the incorporation patterns of carbon atoms into the final molecules [76,83,84,132,133,134,135,136,137]. Additionally, the use of different labelled precursors (i.e., [1-^13^C]-propionate, [methyl-^13^C]-L-methionine, [2-^13^C]-pyruvate, [1-2-^13^C_2_]-succinate, ^13^C-bicarbonate, and [1-^13^C]-glycolate) further clarified the origins of carbon units [45,75,76,132,133]. Among biosynthetic studies, the most extensively investigated classes of APKs include amphidinolides and amphidinols, and more recently, amphidinin A and amphirionin-4 [39,60,137,138].

#### 4.2.1. Labelling Patterns of Amphidinolides

**Figure 7:**
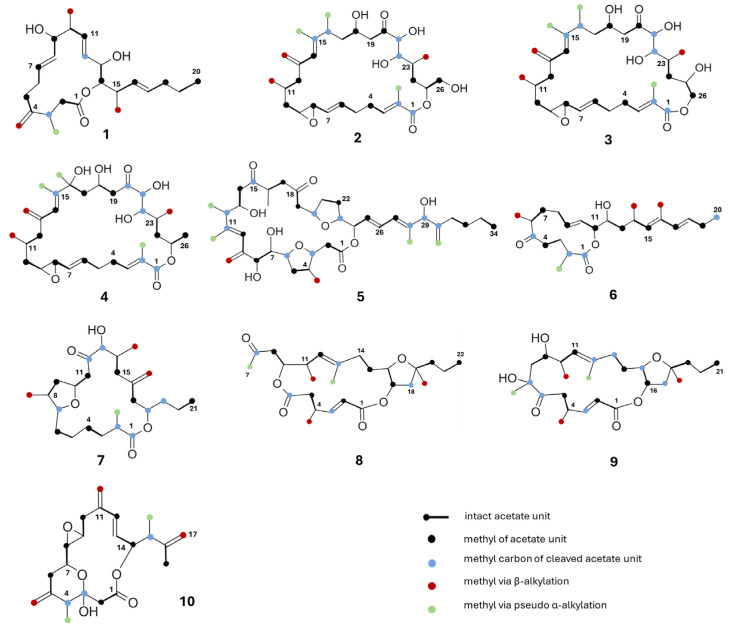
Acetate labelling pattern of amphidinolides J (**1**), H (**2**), G (**3**), B (**4**), C (**5**), W (**6**), T1 (**7**), X (**8**), Y (**9**), and P (**10**).

The first acetate incorporation study was carried out by *Kobayashi* et al. [132] on amphidinolide J (Figure 7, **1**), the most abundant amphidinolide in *Amphidinium* spp. ^13^C NMR analysis revealed a non-successive mixed polyketide origin, consisting of nine intact acetate-derived C_2_ units, two acetate cleavage sites at C3 and C12, and four branched carbons formed through β-alkylation and pseudo-α-alkylation. By contrast, Sato et al. [133] proposed that amphidinolides H (**2**) and G (**3**) originate from three successive polyketide chains disrupted by three unusual “m–m” patterns, derived from acetate methyl groups. These molecules incorporate ten acetate units, six C1 cleavage sites, five methyls, and one methylene group, along with six oxygenated carbons. Amphidinolides H and G share structural similarity with amphidinolide B (**4**), even if notable differences arise in their acetate incorporation patterns. Specifically, the C16–C19 segment of amphidinolide B exhibits a “cm–cm” (carbon–methyl) sequence, while amphidinolide H features a distinct “m–cm–c” (methyl–carbon–methyl) arrangement. Furthermore, amphidinolide B displays an unusual “m–m–m” deletion pattern spanning C20–C22 [135], indicating the absence of three successive acetate-derived methyl groups. Amphidinolide C (**5**) shares similarities with amphidinolide B, particularly on the repeated pattern of two intact acetate units, three deletion steps, and subsequent acetate additions in the C24–C34 region. The C9–C12 segment of amphidinolide C exhibits the same labelling pattern (“c(m)–m–m(m)–m(m)”) found in amphidinolides H and G. However, amphidinolide C is proposed to originate from four diketide chains, incorporating twelve intact acetate units, ten isolated acetate units, and six C1 branches obtained through β- and pseudo-α-alkylation [136]. The isotope labelling pattern of amphidinolide W (**6**) comprises eight intact acetate units, four isolated C1 units, and four branched C1 units that are all suggested to derive from acetates [134]. Kobayashi et al. [76] also examined amphidinolide T1 (**7**) and described a complex biosynthetic pathway involving four polyketide chains, three “m–m” deletion patterns from acetate, an additional cleavage site at C7, and four termina alkylation. Later, Tsuda et al. [84] analysed amphidinolides X (**8**) and Y (**9**), revealing that amphidinolide Y originates from three diketide chains via the incorporation of eight intact acetate units, seven acetate carboxyl deletions, and five C1 branches derived from acetate methyl carbons. More recently, Kubota et al. [137] reported for amphidinolide P (**10**) an origin from seven intact acetate units, three cleaved acetate units, including one “m–m” pattern, and five C1 branches from acetate methyl carbons.

Acetate is the main recognised extension unit in APKs, but alternative precursors have been also investigated. Feeding experiments with labelled pyruvate resulted in a 6% incorporation in amphidinolide H, compared to 8% with acetate, suggesting that pyruvate may be incorporated via its conversion to oxaloacetate [133]. No significant enrichments were observed using labelled propionate or L-methionine, which is consistent with early biosynthetic studies on the amphidinolide J [132]. In order to explain the unusual structure of amphidinolide J (**6**) and understand the involvement of dicarboxylic acids in the process of biosynthesis, the authors also made feeding experiments with labelled succinate. Unexpectedly, no carbon enrichment was retrieved, thus providing no evidence to support the unusual labelling pattern [75,76]. Amphidinolide T1 (**5**) exhibited a markedly different incorporation profile. Feeding with labelled bicarbonate led to a 33% incorporation rate, which is significantly higher than the 3% observed with acetate. This widespread enrichment across the carbon backbone suggests the preferential utilization of carbon dioxide, derived from bicarbonate, in the biosynthetic pathway of amphidinolide T1 [76].

#### 4.2.2. Labelling Studies of Amphidinols and Other Linear Polyketides

**Figure 8:**
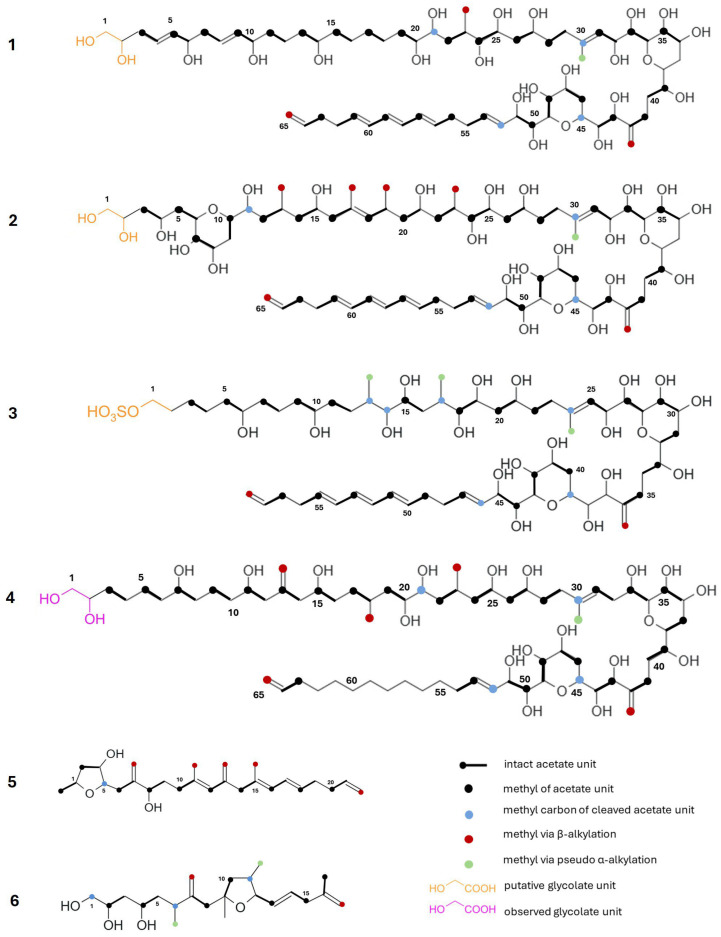
Acetate labelling pattern of amphidinol 4 (**1**), 2 (**2**), 17 (**3**), A (**4**), amphirionine-4 (**5**), and amphidinin A (**6**).

Experiments made using acetate labelling on AM2, AM3, and AM4 demonstrated the role of acetate as a key precursor in the biosynthesis of these amphidinols [138]. The AM4 incorporation pattern suggested sequential acetate additions, followed by a single cleavage event at C21 within its linear polyhydroxy segment (C3–C33). The hairpin loop (C34–C49) retained two methyl groups from acetate cleavage, while an isolated methyl group was found at C52 within the polyolefin region. Additional modifications occurred along the chain, including the β-alkylation of three methyl groups and the pseudo α-alkylation of one methyl group (Figure 8, **1**) [138]. Similar acetate incorporation patterns were observed for AM3, as well as in the polyolefin segment and hairpin loop region of AM2 (**2**), suggesting a conserved biosynthetic pathway. As hypothesised by Houdai et al. [138], other amphidinol congeners (e.g., AM1, AM5 and AM6) could arise from comparable incorporation patterns, while their structural variability can be the result of the recombination of PKS domains. This hypothesis suggests the possible selective activation and/or deactivation of specific modules.

Further insights into AM biosynthesis were provided by the ^13^C-acetate feeding experiments conducted by Meng et al. [48] on AM17 (**3**) and by Cutignano et al. [45] on AM-A (**4**). Specifically, AM17 exhibited significant structural variability in the C3–C27 region, where four carbon deletion steps occurred within the C13–C24 segment. Notably, two of these deletions resulted in the characteristic “m-m-m” labelling pattern commonly observed in amphidinolides [48]. On the contrary, the acetate incorporation pattern of AM-A revealed a structure composed of intact acetate units from C3 to C20, with an interruption at C21 due to the deletion of a single acetate unit. The C21–C53 region showed a strong similarity to AM4 [45]. Interestingly, single- and double-labelled acetate feeding experiments in amphidinols showed no significant enrichment at C1 and C2, suggesting an alternative starter unit.

Following previous observations in dinoflagellate PKs (e.g., okadaic acid), Cutignano et al. [45] identified glycolate as the primary starter unit, with direct incorporation detected at C2 when glycolate degradation via photosynthesis was inhibited. Although this evidence was only demonstrated for AM-A, it could be reasonable to assume that glycolate plays a similar role in the biosynthesis of all AMs.

Further biosynthetic insights have been gained from labelling studies on amphirionin-4 (**5**) and amphidinin A (**6**). Amphirionin-4 (**5**) is derived from non-consecutive acetate extensions, incorporating ten intact acetate units, two irregular C1 units at C5 and C22, and four branched C1 units derived from the C2 carbon of acetate. Its tetrahydrofuran moiety (C1–C6) shares a labelling pattern with the C11–C6 region of amphidinolide T1 (**7**), while the polyene segment (C11–C22) resembles the terminal structural motifs observed in AM2 (**2**), AM4 (**1**), and AM17 (**3**) [39]. In contrast, amphidinin A (**6**) incorporates seven intact acetate units, interspersed with three isolated methyl groups at C1, C6, and C11, and five C1 branches, all originating from cleaved C2 carbons of acetate [137].

## 5. Research Gaps in Biotechnological Applications of APKs

Dinoflagellates of the genus *Amphidinium* produce a variety of long-polyoxygenated PKs and macrolides, which exhibit remarkable activity on tumour cell lines, bacteria, and fungi; thus, they represent potential candidates as new anticancer, antibacterial, and antifungal drugs [11,20,139]. Despite these favourable properties, the limited availability of sufficient quantities has posed significant obstacles in their clinical testing and/or industrialization. While only small amounts are typically required for in vitro and in vivo studies, the transition to preclinical and clinical trials demands significantly larger quantities, underscoring the need for novel strategies to improve the supply chains of these compounds [140,141]. To date, only a few dinoflagellate-derived toxins—such as okadaic acid and saxitoxins—have reached commercial production, with prices varying based on their purity and source. Notably, no compounds isolated from *Amphidinium* spp. are currently available on the market [1]. The absence from the pharmaceutical landscape of a lot of dinoflagellate bioproducts can be attributed to the limited biotechnological use of these species, which present significant challenges compared to other microalgae. General downstream culture processing of *Amphidinium* spp. at a pilot-scale usually involves a first step of centrifugation, after which the biomass is extracted and filtered through several chromatographic approaches (e.g., Sephadex LH-20) to obtain fractions with enriched or pure compounds [139,142]. Challenges include low growth rates, complex metabolic pathways, strict cultivation requirements, and limited biomass yields—typically around 1 g per litre of culture [1,143]. Collectively, these limitations result in reduced yields of bioactive compounds, making large-scale production inefficient [142,144]. Furthermore, the pharmaceutical production of dinoflagellate-derived PKs requires a very high degree of culture purity, adding another layer of complexity to large-scale operations, where maintaining sterile conditions is rarely achievable [20].

Current trends in marine drug biotechnology mainly focus on the improvement of fermentation techniques and the genetic manipulation of microalgal strains [129,145,146,147]. In the case of *Amphidinium* species, efforts to optimise culturing conditions have included the use of various reactor designs (e.g., flat-bottom flasks, plastic cylinders, vertical column and airlift bubble photobioreactors, raceway systems), different operational modes (batch and semi-continuous), and the development of kinetic models to predict growth and production dynamics, such as the mathematical colimitation kinetic model of phosphorus, nitrogen, and light [145,148,149,150,151]. Additionally, the application of nutrient imbalances has been investigated as a trigger for metabolite production. Among culture-based strategies, both abiotic and biotic stress factors have been shown to elicit the biosynthesis of APKs. Abiotic stress methods—such as light variation, salinity shifts, or nutrient limitation—are generally preferred due to their simplicity, reproducibility, and lower operational costs [145,146,152]. In contrast, biotic stresses, which often involve co-culturing with other microalgae or microorganisms, are more complex to manage, less predictable in outcome, and can introduce variables that are harder to control under laboratory or industrial conditions [153,154].

Imbalanced nutrition and targeted supplementation are key aspects in the production of bioactive compounds in *Amphidinium* spp. [145]. The synthesis of AMs, in fact, typically occurs during the late growth phase, and it is strongly influenced by nutrient concentrations and light intensity [145]. Transcriptomic studies revealed that nitrogen deficiency in *A. carterae* and *A. gibbosum* induces no significant changes in the regulation of PKSs [155,156], while phosphorus limitation showed significant effects in the expression of these genes [156]. A similar regulation was also confirmed in another dinoflagellate species, as observed for *Prorocentrum lima* [157]. This response may be attributed to the decreased growth rate under nutrient-limited conditions, which moves the carbon utilisation to the production of defensive compounds. Interestingly, while increased nutrient concentrations have been shown to enhance biomass production in *A. carterae,* they do not affect secondary metabolite biosynthesis [158]. Instead, nutrient surplus led to increased amino acid and sugar production, redirecting metabolic fluxes to specific pathways, namely glutamate−glutamine, GABA, and tricarboxylic acid cycle. However, the same authors have observed that *A. carterae* cultivation exposed to a continuous light cycle (light/dark cycle 24:0) showed an increase in amphidinol A and B production [158]. Other studies have similarly reported that high light intensities induce the production of PKs, because these represent a defence mechanism with respect to light excess [145,159]. In addition, further authors also reported positive effects on the synthesis of PKs in *A. carterae*. For example, Barone et al. [146] investigated the effects of LED blue light (intensity of 100 μmol/m^2^/s), NaHCO_3_ (2.5 mmol/L), salinity (0.8 g/L of NaCl), and H_2_O_2_ (0.5 mM); these conditions increased the production of amphidinols-22, A, B, C, and of other PKS-derived compounds of unclear attribution. In addition to these data, other studies revealed that *A. carterae* in hyposaline conditions increased the production of haemolytic compounds, as was observed for other dinoflagellate species [152,160].

The production of APKs has also been tested by adding different vegetal hormones, such as auxines (indole-3-acetic acid-IAA and 2-naphthoxyacetic acid-BNOA), cytokinins (6-benzylaminopurine-6-BAP, kinetin-KN, and zeatin-ZN), jasmonates (methyl jasmonate-MeJA and jasmonic acid-JA), abscisic acid (ABA), gibberellic acid (GA), and 2-chlorobenzoic acid (CA) [161]. Among these, BNOA, JA, and CA significantly increased haemolytic activity, and, consequently, amphidinol production. Particularly, BNOA (14.84 µM) was found to be the most effective hormone for both small- and large-scale cultures, boosting amphidinols’ yield approximately 4-fold per unit of biomass [161].

Other chemicals, screened as potential stimulators of APKs, include molecules known to induce epigenetic modification, such as inhibitors of the histone deacetylase, DNA-methyl transferase, and others (e.g., 5-azacitidine, hydroxamic suberoylanilide acid, metyrapone, tricyclazole, sodium butyrate, and jasplakinolide). Unfortunately, these compounds induced strong stress to the cells, resulting in growth inhibition and a reduction of photosynthetic yields, without having an impact on the biosynthesis of amphidinols [162].

In addition to abiotic strategies, allelopathy represents a promising strategy to stimulate the production of both known and new secondary metabolites [163]. These approaches often utilise algal cultures. As reported by Kichouh-Aiadi et al. [153], *A. carterae* cultivated in the presence of supernatants of different microalgae (e.g., *Heterosigma akashiwo* and *Pavlova* sp.) induced different responses, including the stimulation of growth and haemolytic activity, but no molecules have been specified so far. Conditions reported to affect APK production are summarised in Table 1.

Although optimising culturing conditions and manipulating nutrient availability have proven effective in enhancing PK production, several critical limitations remain. Among the most significant, there is the need to maintain specific—and often costly—nutritional requirements over prolonged periods, which hinders scalability. To overcome these constraints, synthetic biology and genetic engineering approaches offer promising alternatives [164]. This includes both the direct modification of dinoflagellates and the transfer of their biosynthetic genes into more tractable hosts via customised heterologous expression systems [2,17,122,165,166]. In recent years, traditional approaches have been complemented by advanced technologies, such as genome editing (e.g., CRISPR-Cas systems) and metabolic engineering strategies aimed at increasing precursor supply, removing competing pathways, or redirecting metabolic fluxes [103]. However, the application of these techniques to dinoflagellates remains limited due to their low transformation efficiency and poor genetic characterisation [167].

Dinoflagellates possess unusually large and complex genomes, characterised by high intron density, extensive repetitive non-coding regions, and a permanent liquid crystalline DNA structure enriched in non-canonical bases [22,23,168]. These features severely complicate genetic manipulation. Consequently, most of the current knowledge about the genetic improvement of PK-producing strains derives from bacterial and fungal models, where these techniques have been developed and refined over decades [169,170].

To date, genetic transformation efforts in dinoflagellates have largely focused on enhancing biomass and lipid production rather than PK biosynthesis. In the specific case of *Amphidinium* species, genetic research has primarily explored the introduction of exogenous marker genes, such as β-glucuronidase, to assess their potential as expression platforms, as well as incorporating novel genetic material into the chloroplast genome, thereby laying the groundwork for more targeted genetic manipulation [166,171,172]. Moreover, dinoflagellate genes lack recognisable promoter elements and common eukaryotic transcription factor binding sites [173]. Additionally, in various species—including *Amphidinium* spp.—sophisticated post-transcriptional regulatory mechanisms, such as trans-splicing, have been observed, further complicating the identification of toxin-related genes [126]. This molecular complexity also limits the effectiveness of heterologous expression systems for PKS genes in conventional hosts, such as bacteria, yeast, or mammalian cells, which are typically used as cellular bio-factories. While molecular cloning and recombinant expression have been more extensively explored in other dinoflagellates, applications in *Amphidinium* spp. remain scarce. Notably, recent studies on *Amphidinium carterae* CCMP1314 have reported the successful expression of three different phosphopantetheinyl transferases (PPTases) enzymes that play a critical role in activating PKS and NRPS biosynthetic pathways [174].

Chemical synthesis represents another potential approach, with its own set of advantages and challenges. In the case of APKs, total synthesis has enabled access to several compounds—including various amphidinolides and amphidinols—through highly elaborate routes [175,176]. These syntheses often require between 40 and over 70 individual steps, involving complex fragment coupling, stereoselective transformations, and macrocyclization techniques [175]. While successful in reproducing the natural structures and generating analogues for biological evaluation, such efforts are extremely labour-intensive and economically unfeasible for large-scale production. As a result, chemical synthesis of these molecules is primarily pursued for structural elucidation, mode-of-action studies, and structure–activity relationship investigations, rather than for practical exploitation or commercial manufacturing. Hence, the development of practical and scalable synthetic routes remains a significant hurdle and is unlikely to be achieved in the near future. Omics approaches also represent valuable strategies to study the wide range of metabolites produced by single dinoflagellate species. For example, Osorio-Ramirez et al. [11] recently reported an untargeted metabolomic analysis on *Amphidinium eilatiense,* identifying compounds with biological activities against human cancer cells. Overall, these approaches still do not give definitive responses, considering that this type of study further requires the isolation and the annotation of the identified compounds and specifically tests them against the cell line of interest [11].

## 6. Conclusions

**Figure 9:**
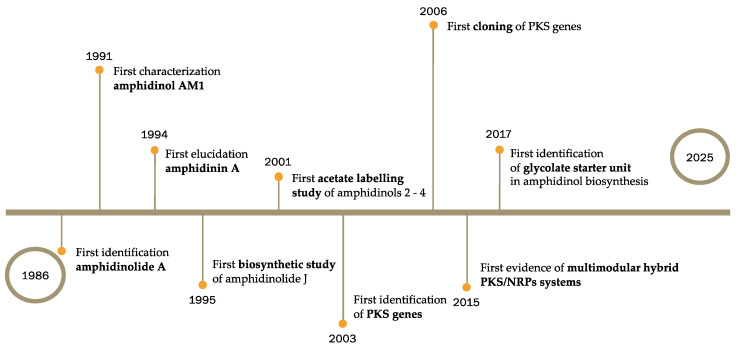
Timeline diagram of the most relevant discoveries concerning *Amphidinium* spp.

This review highlights the extraordinary biosynthetic capacity of *Amphidinium* spp. as prolific producers of structurally diverse and biologically potent polyketides, as discovered in recent decades (Figure 9). A wide array of compounds isolated from this genus, including linear and cyclic members, have demonstrated remarkable pharmacological profiles, such as anticancer, antifungal, and antimicrobial effects. These structurally complex natural products are among the most complex marine secondary metabolites characterised to date.

Despite the promising bioactivity of these compounds, the biotechnological exploitation of *Amphidinium* is constrained by several challenges, including slow growth rates, complex metabolic networks, and limited genetic tractability. Nevertheless, advances in culturing techniques, coupled with the application of high-throughput omics technologies, are gradually beginning to overcome these barriers. Moreover, emerging tools in synthetic biology and heterologous expression offer new opportunities to unlock and optimize the biosynthetic potential of these microalgae, as exemplified by recent synthetic advances toward complex amphidinolide congeners, such as C, F, and U [177].

In light of the growing pharmaceutical interest in APKs, future research should focus on developing scalable culture systems, identifying and functionally characterising key biosynthetic gene clusters, and engineering robust microbial hosts for heterologous expression.

## Figures and Tables

**Table 1:** Laboratory growth conditions reported to positively influence APKs’ production.

Organism	Condition	Dosage	Approach	Molecules	Reference
*A. gibbosum*	Phosphorous starvation	22 µM	Transcriptomic	-	[156]
*A. carterae* BMCC33 (Dn241EHU)	L/D cycle 24:0	-	Metabolomic	Amphidinol A and B	[158]
	Light intensity	573 µE m^−2^ s^−1^	Haemolytic Activity	Amphidinols *	[145]
	Hyposalinity	5 PSU	Haemolytic Activity	Amphidinols *	[152]
	2-naphthoxyacetic acid (BNOA)	14.84 µM	Haemolytic Activity	Amphidinols *	[161]
	2-chlorobenzoic acid (CA)	19.23 µM 192.32 µM	Haemolytic Activity	Amphidinols *	[161]
	Jasmonic acid (JA)	2.38 µM 142.67 µM	Haemolytic activity	Amphidinols *	[161]
	Culture supernatant of *Heterosigma akashiwo*	-	Haemolytic Activity	Amphidinols *	[153]
	Culture supernatant of *Pavlova* sp.	-	Haemolytic Activity	Amphidinols *	[153]
*A. carterae* CCAP 1102/8 (LACW11)	LED blue light	100 μmol/m^2^/s	Metabolomic	Amphidinols A, B, C, 22	[146]
	NaHCO_3_	2.5 mM	Metabolomic	Amphidinols A, B, C, 22	[146]
	Hypersalinity	0.8 g/L	Metabolomic	Amphidinols A, B, C, 22	[146]
	H_2_0_2_	0.5 mM	Metabolomic	Amphidinols A, B, C, 22	[146]

* Hypothetical attribution.

## Data Availability

Not applicable.
